# Biochemical Composition and Seasonal Variations of the Madagascar Algae *Eucheuma denticulatum* (*Solieriaceae*, Rhodophyta)

**DOI:** 10.3390/md23010030

**Published:** 2025-01-09

**Authors:** Elando Fréda Zamanileha, Anne-Sophie Burlot, Thomas Latire, Christel Marty, Philippe Douzenel, Laurent Vandanjon, Nathalie Bourgougnon, Pierre Ravelonandro, Gilles Bedoux

**Affiliations:** 1 Laboratoire de Biotechnologie et Chimie Marines (LBCM), EMR CNRS 6076, IUEM, Université Bretagne Sud, Campus Tohannic, 56000 Vannes, France; elando.zamanihela@outlook.com (E.F.Z.); anne-sophie.burlot@univ-ubs.fr (A.-S.B.); tlatire@uco.fr (T.L.); christel.marty@univ-ubs.fr (C.M.); philippe.douzenel@univ-ubs.fr (P.D.); laurent.vandanjon@univ-ubs.fr (L.V.); nathalie.bourgougnon@univ-ubs.fr (N.B.); 2 Département Procédés et Ecologie Industrielle, Unité de Recherche en Génie de Procédés et Génie de l’Environnement (URGPGE), Université d’Antananarivo, Antananarivo 101, Madagascar; phravelona@yahoo.com; 3 Laboratoire d’Efficacité Cosmétique (E-COS), Université Catholique de l’Ouest Bretagne Nord, 22200 Guingamp, France

**Keywords:** antioxidant, *Eucheuma denticulatum*, Madagascar, monosaccharide, nutrition, seasonal variation

## Abstract

Although the density and diversity of seaweeds in Madagascar is particularly high, these resources are underexploited and they are not part of the local population’s eating habits. No study has been carried out on the nutritional properties and seasonal variation of *Eucheuma* species harvested in Madagascar. In this study, *Eucheuma denticulatum* was harvested monthly over two years (2021 and 2022) on the northeast coast of Madagascar (Sainte Marie Island). The compositional analysis revealed prominent sugars and minerals up to 41.0 and 39.5% dw, respectively. *E. denticulatum* showed slight variability over the seasons in the macroelements and oligoelements (Ca, K, Na, Mg, Fe, Mn) ranging from 22.8 ± 0.2 to 25.3 ± 0.1% dw in 2021 and 22.1 ± 0.3 to 26.5 ± 0.3% dw in 2022. Total amino acids varied from 2.3 ± 0.6 to 2.5 ± 0.6% dw during the two years. Seaweed extracts showed antioxidant activity by the in vitro method ranging from 2026 ± 2 to 2998 ± 4 μg.mL^−1^ in 2021, and from 1904 ± 2 to 2876 ± 4 μg.mL^−1^ in 2022. Finally, the principal component analysis (PCA) showed a correlation between protein content and environmental parameters. The nutritional characteristics therefore confirmed that *E. denticulatum* could potentially be used as a nutritious and functional food and could be incorporated in the diet of local populations.

## 1. Introduction

Over the past three decades, the economic importance of exploiting marine resources has grown, and macroalgae are considered one of the largest biomasses alongside marine crustaceans and freshwater fishes [1,2]. Marine organisms are also potentially productive sources of bioactive metabolites, which could provide useful leads for the development of new pharmaceutical agents [1,2,3]. These activities are therefore perfectly in line with Sustainable Development Goal 14, which aims to conserve and sustainably use the oceans, seas and marine resources [4]. In order to move closer to this goal, the exploitation of algae has been favored worldwide. Around 80% of harvested seaweed is destined for direct and indirect human consumption. Non-food applications include the production of animal feed, pharmaceuticals, hydrocolloids, cosmetics, fertilizers, cosmeceuticals, biostimulants and bioactive compounds [5,6].

Marine macroalgae are photosynthetic organisms typically classified by their pigmentation as brown, red or green [7,8,9], and are produced from two sources: wild and/or cultivated stocks [5]. According to FAO statistics in 2019, 35.8 million tons of the world’s seaweed production was produced by 54 countries/territories, with 97% of production coming from cultivation [10,11,12,13,14]. Commercially cultivated macroalgae include species from 17 genera, of which *Eucheuma*, *Gracilaria*, *Hydropuntia*, *Hypnea*, *Kappaphycus*, *Cladosiphon* and *Caulerpa* are mainly grown in tropical and subtropical areas [13,15]. The high value and wide applications of *Kappaphycus* and *Eucheuma* have prompted renewed efforts over the past three decades in areas such as molecular systematics, selection of better strains to increase yields, refinement of cultivation and management systems and development and marketing of value-added products [10,11].

Macroalgae not only contain large quantities of dietary fibers, proteins and polysaccharides, but also a variety of functional polyphenols, antioxidants, pigments and lipids [12,13,14,16]. Biochemical composition can vary according to species, geographical location, season and temperature [16]. Macroalgae contain minerals and trace elements, such as sodium, calcium, chlorine, magnesium, zinc and copper. The protein content of algae is generally high; this is particularly true of red algae, where protein can reach 40% of dry matter [17,18,19]. In addition, storage polysaccharides (starch, laminarin) are diverse [16]. Red algae (Rhodophyta) have a higher protein content than green or brown algae [12,20], making them interesting from a nutritional point of view [20]. They are also a source of vitamins, particularly vitamin C and members of the B family [18,21,22]. The nutritional values of certain red algae have been well documented in recent studies [23,24,25], and much evidence has been gathered indicating that these marine organisms could hold promise for functional foods. Antioxidants are compounds capable of inhibiting reactive oxygen and free radicals in the body by donating one or more electrons to free radicals [26,27]. Through the importance of antioxidants, it has been proven that there is a positive correlation between the amount of antioxidants in the diet and the reduction of cancer mortality in an individual [28,29,30,31,32]. Studies have shown that red algae have a high potential for antioxidant activity [16,27,28,29].

The *Kappaphycus* and *Eucheuma* algae are primarily cultivated in Southeast Asia, Tanzania and Kenya, with modest production in Latin American countries. In 2022, WoRMS listed 23 species of *Eucheuma*, while AlgaeBase recorded 26 species [31,32]. This red algae genus is a significant source of iota-carrageenan, used in the food, pharmaceutical and manufacturing industries. It supports small-scale industries in coastal regions of Southeast Asia, the Western Pacific and the Indian Ocean [33]. In 2019, *E. denticulatum* production reached 180,000 tons, mainly in Zanzibar and the Philippines, a decrease from 2015 levels [34]. *E. denticulatum* remains a key species for iota-carrageenan production, intensively cultivated in these regions to meet global demand [35]. Recent studies have explored the nutritional composition of red algae, highlighting their richness in vitamins (A, B, C), proteins, lipids, carbohydrates, dietary fiber, minerals and essential amino acids. The species studied show significant levels of vitamins B1, B2 and C [36]. Others have shown that the *Eucheuma* species reveals a complete profile of essential amino acids and minerals as well as carbohydrates, with low levels of lipids [37]. These results underline the potential of algae for health and the fight against malnutrition.

Madagascar is considered one of the richest countries in terms of biodiversity, with a high rate of endemism that is proving to be a global priority [38,39]. It is one of the main biodiversity hotspots in the western Indian Ocean, but, as in many other regions, the coral reefs surrounding the island are facing large-scale disturbance and local human-induced stressors [40]. Looking back in history [41], in 1989, the IH.SM of Toliara (Institut Halieutique des Sciences de la Mer) initiated seaweed cultivation at Songeritelo, located in south-west Madagascar. The first cultivation trials took place and continued, with ups and downs, for several years. In 1997, the launch of the FED-ARPL project, in collaboration with IH.SM and the BioMed Company in the province of Toliara, Madagascar, gave a new start to seaweed cultivation. In 1998, a variety of *Eucheuma striatum* was imported from Zanzibar to take advantage of an algae specifically chosen for cultivation, superior in resistance and growth to local strains. The project was then extended to Nosy Be, in the northwest, and Nosy Ankao, north of Vohemara, in the northeast. This led to the creation of the second IBIS red algae company in northern Madagascar in 1998. Since 2015, *Eucheuma denticulatum* has only been cultivated by IBIS in Ampasimadera (northern Madagascar), with an annual production of 300 tons dry [42], but currently this seaweed cultivation has expanded to the Eastern Cape. More than five years later, in 2020, production of dry seaweed, mainly *E. cotonii* and *E. spinosum* species with 10% dry seaweed (dw), reached 2300 tons [41]. There are currently seven major commercial operators in this market, six of which started operating in 2021 [41].

In Madagascar, *Eucheuma denticulatum* (Burman) Collins et Hervey (Rhodophyta, Solieriaceae), known as “spinosum”, is widespread along the 300 km of coastline between Toliara and Morondava [43]. This species is known for its main source of iota-carrageenan, which has been cultivated in the country for 50 years. This species is currently being cultivated and is slowly spreading along the east coast. Madagascar invests little in its health sector compared with other countries, where per capita expenditure is USD 20, whereas the WHO recommends USD 34 [44]. As a result, Madagascar’s nutritional situation is precarious, and chronic malnutrition is a public health and development problem. According to UNICEF, in 2023, Madagascar was one of the 10 most malnourished countries in the world, with 47% of children under 5 (around 2 million children) affected, mainly in rural areas [45]. Faced with the problem of malnutrition in Madagascar, *Eucheuma* species, which grow in certain regions, could be introduced in the human diet and contribute to the Malagasy economy.

The data on biochemical composition of *Eucheuma denticulatum* species from Madagascar lack as well as the environmental abiotic parameters of marine areas. So, this study focuses on the analysis of seasonal variations of algal growth areas and of algal biochemical components. Among the nutritional compounds, this work presents the contents of amino acids by chromatography, and of minerals by atomic absorption spectrophotometry. The monosaccharides of iota-carrageenan were determined with anion exchange chromatography. These analyses will provide a deeper understanding of the nutritional and economic potential of *Eucheuma denticulatum*, paving the way for solutions to combat malnutrition while promoting the sustainable use of local marine resources.

## 2. Results

### 2.1. Follow-Up of Environmental and Abiotic Factors

**Figure 1:**
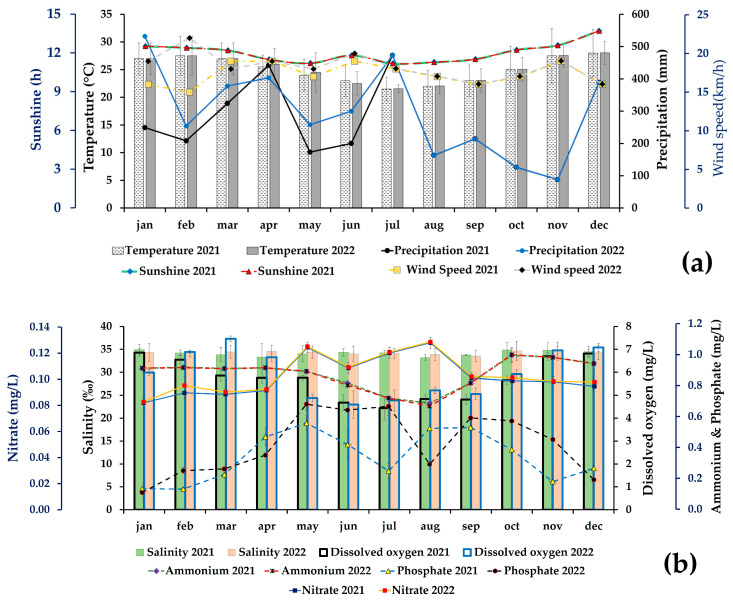
Follow-up of variations in environmental and abiotic factors through variations in temperature, sunshine, wind speed and precipitation (**a**) and variations in seawater salts (salinity, nitrate, ammonium, dissolved oxygen and phosphate) (**b**), years 2021 and 2022 (summer season October–January and winter season May–September).

Physical factors (temperature, sunshine duration, wind speed and precipitation) and chemical parameters (salinity, dissolved oxygen, nitrate, ammonium and phosphate) were recorded at each collection in order to monitor the alga’s behavior in relation to climate change. The seasonal variations over the years 2021 and 2022 are presented in Figure 1. The results showed that temperature, sunshine duration, precipitation and wind speed have similar seasonal profiles. Mean temperatures between 2021 and 2022 were 25 ± 3 °C and 25 ± 2 °C, respectively, while sunshine duration was 12.0 ± 0.8 h, and wind speed 17 ± 1 km.h^−1^ and 18 ± 2 km.h^−1^. Total precipitation in 2021 was 3049.0 mm and 3578.2 mm in 2022. Mean salinity was constant: from 2021 to 2022, it was 34 ± 1 and 34 ± 2 ‰. Dissolved oxygen levels averaged between 5.7 ± 0.2 mg.L^−1^ (year 2021) and 6.0 ± 0.2 mg.L^−1^ (in 2022). On the other hand, seawater quality varied with the seasons with the following maximum and minimum values in 2021: nitrate ranged from 0.082 ± 0.002 mg.L^−1^ to 0.128 ± 0.001 mg.L^−1^, ammonium from 0.69 ± 0.04 mg.L^−1^ to 1.00 ± 0.04 mg.L^−1^ and phosphate from 0.13 ± 0.01 mg.L^−1^ to 0.56 ± 0.05 mg.L^−1^. In 2022, nitrate and ammonium concentrations differed little from the seasonal variation, with minimum and maximum values ranging from 0.083 ± 0.005 mg.L^−1^ to 0.129 ± 0.004 mg.L^−1^ and 0.67 ± 0.01 mg.L^−1^ to 1.00 ± 0.05 mg.L^−1^, respectively, while phosphate concentrations differed considerably, from 0.11 ± 0.06 mg.L^−1^ to 0.68 ± 0.04 mg.L^−1^.

### 2.2. Biochemical Composition of Eucheuma Denticulatum

The results of the biochemical composition of *E. denticulatum* are presented in Table 1 and expressed in percentages relative to dry weight (% dw). In 2021, they ranged from 20 ± 7 to 29 ± 2% dw ash, from 28.72 ± 0.01 to 41.0 ± 0.4% dw of neutral sugars, from 1.262 ± 0.001 to 3.08 ± 0.01% dw of uronic acids, from 3.7 ± 0.1 to 14.5 ± 0.3% dw of sulfate groups, from 4.8 ± 0.8 to 7.3 ± 0.7% dw of proteins and from 7.344 ± 0.001 to 12.65 ± 0.01% dw of 3,6-anhydrogalactose, the total amount of which varies from 69.8 ± 1.5 to 90.3 ± 2.4% dw of dry matter. In 2022, total biochemical compound contents ranged from 72.4 ± 2.5 to 98.7 ± 5.1% dw, with *E. denticulatum* dried powder varying from 23.7 ± 0.4 to 39.5 ± 0.3% dw ash, 28.1 ± 0.1 to 38.7 ± 0.1% dw of neutral sugars, 1.142 ± 0.001 and 2.44 ± 0.01% dw of uronic acids, 3.71 ± 0.06 and 9.86 ± 0.25% dw of sulfate groups, 1.02 ± 0.24 to 6.29 ± 0.42% dw of proteins and 7.75 ± 0.01 to 11.655 ± 0.001% dw of 3,6-anhydrogalactose. Statistical analysis reveals that each sample tested is significantly different from the others, which was verified by ANOVA tests with a significance level below 0.05 (*p*-value < 0.05).

### 2.3. Extraction and Characterization of Carrageenans from Eucheuma Denticulatum

#### 2.3.1. Extraction of Carrageenans

The results obtained using this method are comparable to those reported in the article published by Vandanjon et al. in 2023 [46]. In general, extractions yielded carrageenan levels in excess of 50% dw of the starting dry seaweed between 80 and 90 g. In fact, the extractions achieved carrageenan yields in excess of 50% of the total volume (Table 2). Yields vary according to the year and season of collection. In 2021, the maximum yield is 61.7% dw and the minimum yield is 53.7% dw. The maximum value was raised to 66.0% dw in 2022, while the minimum decreased to 53.1% dw. Statistical analysis results are similarly significant in terms of yield (*p* < 0.05), with relatively limited variation between the carrageenans obtained.

#### 2.3.2. Monosaccharides’ Composition

The composition of monosaccharides in carrageenan extracts was determined by high-performance anion exchange chromatography (HPAEC). Results were expressed in percentage of total sugar content (% TS). Of the ten monosaccharides identified, the main contents were galactose, which accounted for over 60% TS in each sample, followed by glucose, glucuronic acid and xylose. Smaller amounts of rhamnose, arabinose and glucosamine were found, as well as traces of ribose, fructose and mannose. All these monosaccharides were present in every sample and showed significant differences varying according to the season. The content of total monosaccharides varied from 74.2 ± 4.4 to 86.3 ± 5.4% TS based on the dry matter of the algae studied (Table 3). During the year 2021, the total monosaccharide content was in the range of 77.6 ± 4.6 to 82.7 ± 2.7% TS, respectively, for the months of December and August from minimum to maximum. The contents in December (summer) corresponded to 47.1 ± 4.3% TS of galactose, 2.5 ± 0.1% TS of glucose, 1.3 ± 0.1% TS of glucuronic acid, 1.01 ± 0.03% TS of xylose and less than 0.5% TS of arabinose, rhamnose and glucosamine. Thus, the maximum value in August (winter) comprised 49.5 ± 2.4% TS of galactose, 2.6 ± 0.3% TS of glucose, 1.3 ± 0.1% TS of glucuronic acid and 1.0 ± 0.1% TS of xylose. Concerning the year 2022, these quantities reached 74.2 ± 4.4 and 86.3 ± 5.4% TS, corresponding to the months of October and November. For the minimum content in October 2022, the values consist of 43.2 ± 3.6% TS of galactose, 2.4 ± 0.5% TS of glucose, 0.6 ± 0.2% TS of glucuronic acid and 1.0 ± 0.2% TS of xylose. In November, the content of monosaccharide elements was therefore 55.2 ± 4.7% TS of galactose, 2.7 ± 0.3% TS of glucose, 1.5 ± 0.2% TS of glucuronic acid and 1.2 ± 0.2% TS of xylose.

#### 2.3.3. FTIR Analysis of Carrageenan Extracts

**Figure 2:**
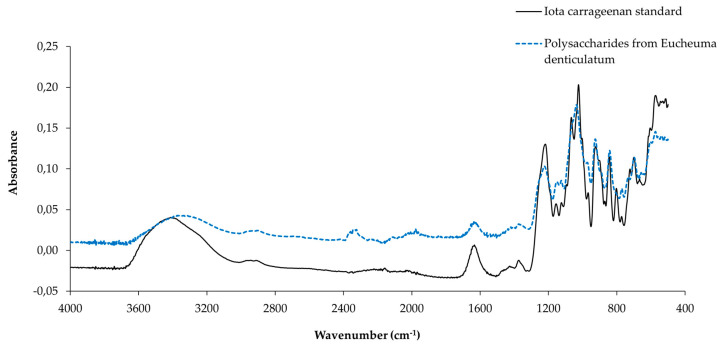
Extract of FTIR spectra of standard iota carrageenan and *Eucheuma denticulatum* polysaccharides collected in January 2022.

In red algae, the polysaccharide is characterized by its presence on the cell wall as a defense mechanism. In this region, where algae are at the mature stage of their development cycle, so that the cell wall is relatively rigid, highly viscous and offers considerable protection, they generate mainly iota-type carrageenans [46]. According to the article by Vandanjon et al. 2023 [46], during the mature growth phase of *Eucheuma denticulatum*, iota-type carrageenan precursors can be detected by FTIR spectra. This FTIR spectroscopic analysis of the extracted polysaccharides (Figure 2) confirmed that carrageenans and galactans (galactose polymer) were the main matrix polysaccharides of *E. denticulatum* collected in January 2022. In the article by Vandanjon et al. 2023 [46], we discussed the analysis of this spectrum, where peaks are attributed to the following bands.

The ester-sulfated 1221.5 cm^−1^ (S=O) is identified as ι-, κ- and ν-carrageenan [16,47]. C-O of 3,6-anhydrogalactose (DA: 4-Linked 3,6-anhydro-α-D-galactopyranose) is identified for ι- and κ-carrageenan from 1061.82 cm^−1^ (shouldered). Next, the 973. 16 cm^−1^ band (shouldered peaks) contains galactose (G/D: 3-Linked β-D-galactopyranose/4-Linked α-D-galactopyranose) as well as ι-, κ- and ν-carrageenan. Subsequently, the 925. 06 cm^−1^, C-O band of 3,6-anhydrogalactose (DA: 4-Linked 3,6-anhydro-α-D-galactopyranose) is specific to ι-carrageenan. Then, at a low intensity of 905 cm^−1^ (thickness), C-O-SO_4_ (group ester) is used on the C_2_ of 3,6-anhydrogalactose (DA2S: 4-Linked 3,6-anhydro-α-D-galactopyranose 2-sulphate) for the ι- and κ-carrageenan-specific band. Next, C-O-SO_4_ is used on C_6_ galactose (G/D6S: 3-Linked β-D-galactopyranose/4-Linked α-D-galactopyranose 6-sulphate) for ν-carrageenan. In total, 843 cm^−1^ C-O-SO_4_ is used for C_4_ galactose (G4S: 3-Linked β-D-galactopyranose 4-sulphate) for ι-, κ- and ν-carrageenan, while 805 cm^−1^ C-O-SO_4_ intensity is used for 3,6-anhydrogalactose C_2_ (DA2S: 4-Linked 3,6-anhydro-α-D-galactopyranose 2-sulphate) specific for ι-carrageenan. According to this analysis, the bands are also similar to those of standard iota. Thus, given that, this study concurs with the article published by Vandanjon et al. 2023 in the part of the study of *E. denticulatum*, whereby it was found that when the alga is at the juvenile stage, traces of kappa-carrageenan and nu-carrageenan polysaccharides in some extracts are mixed, but at the mature stage, the majority carrageenan is of the iota type [46].

### 2.4. Mineral Composition of Eucheuma Denticulatum

**Figure 3:**
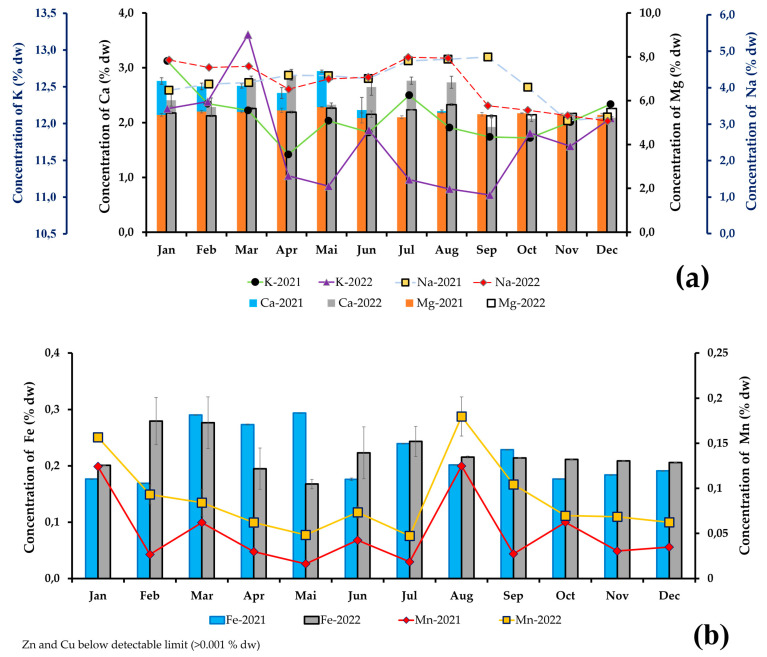
Mineral composition in *Eucheuma denticulatum* from Madagascar years 2021 and 2022 in % dw: (**a**) macroelement composition and (**b**) oligoelements composition.

Mineral contents were expressed in grams per dry weight of algae of total mineral matter (% dw). The analytical results showed a high total content (Figure 3), since in 2021, the maximum macroelement content was 25.0 ± 0.1% dw and the minimum 22.6 ± 0.2% dw (in May and November, respectively); potassium had the highest content between 11.6 and 12.8% dw, followed by magnesium (5.2–5.7% dw), then sodium (3.1–4.8% dw) and calcium (2.0–2.9% dw). The content of oligoelements was also high, ranging from 0.20 ± 0.01 to 0.35 ± 0.01% dw (in February and March, respectively); and included iron (0.17 and 0.30% dw), manganese (0.02 and 0.13% dw) and lower levels of zinc and copper, less than 0.001% dw. In 2022, the mineral composition was rich in macronutrients, representing a total of 26.2 ± 0.2 and 21.8 ± 0.3% dw (in the months of September and March) of which, potassium had the highest content (11.0–13.2% dw), followed by magnesium (5.3–5.8% dw), sodium (3.1–4.8% dw) and calcium (1.9–2.9% dw). Oligoelements ranged from 0.20 ± 0.01 to 0.35 ± 0.05% dw (in May and February), containing 0.17–0.28% dw of iron, 0.01–0.08% dw of manganese as well as zinc and copper (<0.001% dw).

### 2.5. Amino Acid Composition of Eucheuma Denticulatum

**Figure 4:**
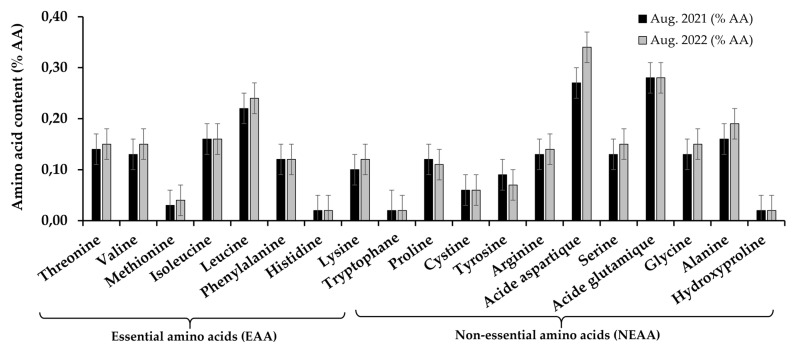
Amino acid profile of *Eucheuma denticulatum*, expressed in (% AA) dry matter of total amino acids detected by LC.

Referring to the two samples analyzed for *E. denticulatum* algae harvested in August 2021 and 2022, the amino acid profile of the selected algae showed some similarity but also large differences in the concentrate ions of certain amino acids (Figure 4). Results are expressed in relative percentage of algae dry weight in amino acids (% AA). The analyses show that there are no substantial differences in the amino acid profile of the two samples analyzed. Total essential amino acids (∑EAA) in these collection years range from 0.94 ± 0.25 to 1.02 ± 0.24% AA (August 2021 and 2022, respectively), including 0.14 and 0.15% AA threonine, 0.13 and 0.15% AA valine, 0.03 and 0.04% AA of methionine, 0.16% AA each isoleucine, 0.22 to 0.24% AA of leucine, 0.12% AA of phenylalanine, 0.10 and 0.12% AA of lysine, 0.02% AA of tryptophan and less than 0.02% AA of histidine. In the non-essential amino acid class, the total content (∑NEAA) is 1.55 ± 0.33 and 1.66 ± 0.33% AA (August 2021 and 2022, respectively), made up of aspartic acid (0.27 and 0.34% AA), glutamic acid (0.28 and 0.28% AA), alanine (0.16 and 0.19% AA), arginine (0.13 and 0.14% AA), glycine (0.13 and 0.15% AA), serine (0.13 and 0.15% AA), proline (0.12 and 0.11% AA), tyrosine (0.09 and 0.07% AA), cystine (0.06 and 0.06% AA) and hydroxyproline (less than 0.20% AA). Total amino acid contents (∑AA) varied between 2.46 ± 0.55 and 2.68 ± 0.54% AA (in August 2021 and 2022, respectively). Thus, the ratios of each amino acid class to total amino acids (∑AA) are as follows: for the ratio of Σ EAA/Σ AA is 40.34% and 40.32% (2021 and 2022, respectively), for Σ NEAA/Σ, AA is 59.66% (2021) and 59.68% (2022) and the ratio between the essential and non-essential amino acid class (Σ EAA/Σ NEAA) is 67.63% and 67.55% (in 2021 and 2022, respectively).

### 2.6. Antioxidant Activity of Carrageenan

The free radical scavenging capacity of *carrageenan* extracts was estimated by reducing DPPH radical scavenging. DPPH is scavenged by antioxidant molecules via hydrogen uptake to form reduced diphenylpicrylhydrazine (DPPH-H), thus neutralizing the reactive chain and reducing the risk of oxidative damage to cells [29,48,49,50]. Butylated hydroxyanisole (BHA) and butylated hydroxytoluene (BHT) standards showed inhibition concentrations (IC_50_) of 8.2 ± 0.7 and 13.5 ± 0.6 μg.mL^−1^, respectively. Table 4 shows the maximum antioxidant activity efficiencies of the extracts in percent, and the inhibition activities (IC_50_) are expressed in µg.ml^−1^. In 2021, inhibition activities ranged from 2026.5 ± 1.2 to 3050.2 ± 2.2 μg.mL^−1^ (corresponding to the months of August and February). In the harvest year 2022, this value ranged from 1904.0 ± 1.1 to 2875.6 ± 2.0 μg.mL^−1^ (in the same month of 2021). In both years, there were few significant differences between seasons. The IC_50_ values observed for 2021 and 2022 are generally similar, but significant variations are observed from one year to the next, depending on environmental conditions.

## 3. Discussion

### 3.1. Environmental and Abiotic Factors for Algae Growth

The introduction of non-native strains of *E. denticulatum* into the western and tropical Indian Ocean has led to its spread into the surrounding seascape [51]. The study conducted by Kimathi et al. (2018) at the Kibuyuni cultivation sites in Kenya, where *E. denticulatum* grows extensively, showed that under normal conditions, the minimum and maximum temperatures of seawater were between 27.8 ± 0.3 °C and 30.5 ± 0.4 °C, a salinity of 35.4 ± 0.1‰, a nitrate content of 1.3 ± 0.2 µmol.L^−1^ and phosphates of 0.7 ± 0.1 µmol.L^−1^ [51]. These conditions correspond to the range of parameters of our study. Nevertheless, maintaining a balance between the natural state and water quality may be crucial for the growth of *E. denticulatum.* Coastal bays are affected by a number of environmental factors that reflect the interactions between the coastline and the ocean [52]. Natural variations, human activities and oceanic changes affect these ecosystems [53]. *E. denticulatum* production is seasonally variable compared with cultivated areas, but growth rates at the site (wild culture) were high throughout the year and similar to those of cultivated algae in the coastal sea of Sainte Marie Island such as temperature, salinity, wind speed and sunshine duration which have similar seasonal profiles. The island has a tropical climate with two main seasons: a hot, humid summer season (October–January) and a cooler, drier winter season (May–September). Winds blow at low speeds, with trade winds east of the site (2021–2022). The results suggest a range of environmental values for which good eucheumatoid growth can be expected. These conditions favored high growth rates of the alga *E. denticulatum* [54]. Variations in environmental salinity caused by evaporation play a crucial role in algal growth and biomass quality. Different salinities have been shown to have an impact on algal growth and levels of lipids, proteins and carbohydrates [55]. Precipitation therefore impacts in transporting nutrients to the oceans. Nitrogen concentration leads to an increase in algal nutrient richness, while precipitation leads to a decrease in biomass [56]. Algal physiology is also influenced by changes in salinity, temperature, hydration and light levels during the water cycle and the seasons [57].

Dissolved oxygen levels did not vary greatly over the two years of the study, but varied somewhat between seasons. Summer concentrations ranged from 6.0 ± 0.2 to 7.5 ± 0.1 mg.L^−1^ and winter levels from 4.5 ± 0.5 to 5.2 ± 0.1 mg.L^−1^. In addition to the non-conservative properties of dissolved oxygen, ammonium, nitrate, nitrite, phosphate and silicate respond to biogeochemical processes [58,59]. This is shown by the variations in nitrate, phosphate and ammonium concentrations in Figure 1b. Independently measured, concentrations of nitrogen compounds such as nitrates range from 0.10 ± 0.03 to 2.00 ± 0.03 mg.L^−1^ during the summer season. In winter, concentrations range from 2.00 ± 0.02 to 2.07 ± 0.02 mg.L^−1^. These concentrations were classified at the intermediate quality concentration standard for the coastal zone, which lies between (10.0 and 50.0 μmol.L^−1^) [60,61]. For ammonium, the average concentration over the summer season is 0.91 ± 0.01 mg.mL^−1^ and 1.00 ± 0.05 mg.mL^−1^, values that are classified as high quality waters. Like nitrates, nitrites and ammonium stimulate the growth of plankton and aquatic grasses that support higher trophic levels [62]. Similarly, phosphate concentration is also high, ranging from 0.13 ± 0.01 mg.mL^−1^ to 0.572 ± 0.003 mg.mL^−1^ in summer, and from 0.25 ± 0.04 mg.mL^−1^ to 0.70 ± 0.04 mg.mL^−1^ in winter. Thus, in relation to these concentration variations, winter mixing caused by wind and rain also injects nitrates, as well as phosphates and silicates, into the euphotic zone from deeper waters [63]. Studying the concentrations of nitrogen compounds in seawater is useful for identifying the intermediate oxidation state between ammonium and nitrate. It can also represent specific anthropogenic inputs and help identify hazards of little concern to public health. However, it is also nitrogen compounds that have the greatest toxicological significance for human health. They are present in reasonable concentrations in the diet [64].

In general, the plant kingdom is exposed to abiotic stress that causes a variety of disorders, but marine algae have defense mechanisms to cope with this stress [65]. The growth of algae is mainly due to the presence of large quantities of nitrogen and phosphorus in the water [66]. These nutrients are leached from soils and farmland containing large quantities of nitrogen and phosphorus fertilizers. Pollution of waterways, in particular the dumping of untreated or poorly treated industrial waste, leads to the release of raw toxic waste into aquatic ecosystems. The result is dense algal growth [67]. The data obtained provide a better understanding of the environmental consequences of the growth of the alga *E. denticulatum* on coral reefs, and underline the importance of maintaining viable populations of macroalgal-feeding fish in these areas. In addition, the influx of freshwater reduces salinity and dissolved oxygen. The osmolarity of seawater changes (hypo-osmotic environment) [68]. Sunshine hours are longer than in other seasons, and seawater salinity and nitrate and ammonium content increase. Algae continue to grow and reach larger sizes, cell wall synthesis remains active and cells elongate [69]. In summer, the seawater of the island of Sainte Marie is also characterized by the presence of chlorophyllous organisms (potential competitors or light aggressors) [67,70,71]. Small compounds with nitrogenous properties can also be synthesized, playing a role in the algae’s light protection, defense and repair strategies [67,72,73]. The respective contents of each metabolite in algae vary greatly depending on season, state of growth, geographical area and environment quality.

### 3.2. Biochemical Composition

#### 3.2.1. Content of Neutral Sugars, Uronic Acids, Total Sulfate and 3,6-Anhydrogalactose

The biochemical composition of *E. denticulatum* harvested on Sainte Marie Island in Madagascar showed a high content of neutral sugars and ash. The neutral sugar content (26.3 to 41.0% dw) is slightly higher than that of red algae, compared with the study carried out by Bedoux et al. 2017 with 15.6 ± 0.8% dw of *Solieria filiformis*. This higher sugar content enables it to withstand various biotic and abiotic stresses [73]. The high content of neutral sugars corresponds to the synthesis of carrageenan and osmotic regulators [74]. The carbohydrates stored in red algae consist mainly of floridean starch. The uronic acid content ranged from 1.1 to 3.1% dw in the two collection years 2021–2022 (see Table 1). Thus, glucuronic acid is known to be a minor component of several sulfated galactans, particularly DL-hybrids, where it typically forms single branches on the galactan backbone [75]. The 3,6-anhydrogalactose ranged from 7.3 to 12.7% dw (Table 1) as a component of iota-carrageenan [76]. It was found that in *E. denticulatum*, the content of sulfate groups varied from 3.7 to 14.5% dw with variation depending on the season; these values are similar to studies carried out on other red algae in articles published by Burlot et al. (2023) and Bedoux et al. (2017), which ranged from 10.0 to 14.5% dw and 7.5% dw, respectively, for algae of the species *Solieria chrodralis* and *Solieria filiformis* [75,77]. This may be due to the persistence of sulfates that bind to the carrageenans of red algae [73].

In summer (December to March), when *E. denticulatum* from Madagascar reaches maturity, it contains a majority of iota-type carrageenan polysaccharides. The total sulfate content is therefore higher in this season [46]. In winter (June to September), the seaweed is in a growth phase when the plant tillers develop and grow, so 3,6-anhydrogalactose levels are high and the amount of carrageenan also increases. Carrageenans thus contribute to the strength of the algal cell wall. The importance of the matrix phase in red seaweed stems from the specific properties of the marine environment (resistance to current, osmotic pressure, ion exchange, water retention at low tide) [78]. It adapts its various defense mechanisms to the imbalance of the seaweed [46]. They still contain a lot of floridean starch, whereas previously they released thallus. In addition, salinity increases in summer, mainly due to water evaporation resulting from the longer photoperiod at this time (hyperosmotic environment). In response to this osmotic pressure and to prevent cell plasmosis, algae synthesize and accumulate osmoregulatory compounds such as fluoridosides [79]. Seasonal variations then influence the biochemical and biological characteristics of the compounds, which in turn influence the structure of the sulfated galactans [27,80,81,82]. They play an important role in protecting the thallus against biotic and abiotic stresses in its environment.

#### 3.2.2. Total Protein Content

According to Table 1, the protein content of *E. denticulatum* during the summer seasons of 2021 was lowest (5.1 ± 0.2% dw), while the highest level (7.0 ± 0.7% dw) was in April. In 2022, the lowest level was 2.04 ± 0.64% dw in December, and the highest was 6.3 ± 0.4% dw in January. Comparing these values with the study carried out by Anne-Sophie Burlot’s thesis in 2016, they are lower than the alga *Solieria chodralis* from Brittany in France, whose average protein content was 14.5 ± 2.0% dw [83], and also lower than the article published by Matanjun et al. (2009) with 9.76 ± 1.33% dw from *Eucheuma cottonii* [37]. In winter, for the year 2021, contents vary from 4.76 ± 0.83 to 7.31 ± 0.72% dw in May and September, respectively, which is higher than the study average. In 2022, it ranged from 3.06 ± 2.16 to 3.05 ± 4.82% dw (in September and August, respectively). In contrast, the protein content of *E. denticulatum* studied corresponds perfectly to that of red algae (2–8% of dry weight) [84]. This value is close to that of the protein content of *Palmaria palmata* (Rhodophyta) [85,86], *Fucus* sp (*Phaeophyceae*) [87] and *Solieria chrodalis* (Rhodophyta) [88]. The protein content of *E. denticulatum* is comparably lower than that of protein-rich plant foods such as soya, so the values obtained correspond to legume foods [89,90]. Variations in the protein content of seaweed can occur according to species and season [91,92]. This phenomenon can also be due to precipitation, which causes soil leaching, as well as to large quantities of fresh water loaded with nitrogenous nutrients, such as nitrate and ammonium, from rivers. Algae then assimilate the abundant nitrogen to produce proteins such as phycobilliproteins [46,93], which are needed to capture light energy, which is reduced at this time of year.

#### 3.2.3. Mineral Composition

Algae are traditionally valued for their high mineral concentration, as they contain more essential elements than terrestrial plants [17,94]. In general, the results concur with information in the literature concerning the mineral composition of red algae [17,24,25]. According to the results of this study, the *E. denticulatum* species has a high content of essential elements (macro-elements) in the order of 21.769 and 26.243% dw, with a high concentration of minerals such as potassium and sodium. Oligo-elements range from 19.58 to 35.22% dw. The ash content ranges from 20.237 to 39.49% dw (Table 1), indicating that *E. denticulatum* contains a high quantity of mineral elements. The biomass of this alga can be considered one of the most promising for the utilization of various macroelements, as it has been shown to have a balanced mineral composition. Certain stages of photosynthesis are catalyzed by minerals. It is conceivable that their main atoms are combined with organic compounds essential to life [95]. In addition to the macro-nutrients mentioned above, red algae also contain a variety of nutrients present in very low concentrations, but which have a significant beneficial effect on human health. The percentages of the Recommended Daily Allowance covered by consuming 8 g of dried seaweed are also given, as is the Recommended Daily Allowance [18]. The majority of macronutrients present in red algae are sodium, potassium, magnesium, phosphorus and calcium. According to the study by Matanjun et al. (2009), *Eucheuma cottonii* species are widely studied in the Kenya; we find 1.77% dw of sodium, 13.16% dw of potassium and 0.27% dw of magnesium [37]. The mineral content of *E. denticulatum* from Madagascar is therefore lower than that of *Eucheuma cottonii* from Kenya. The importance of minerals is crucial to the survival of living organisms such as algae. To meet their physiological needs, algae require water, light and minerals, both ionic and non-ionic. Consequently, the mineral content of *E. denticulatum* varies according to the season, with summer having a higher mineral content than winter.

#### 3.2.4. Characterizing Carrageenans and Monosaccharide Content

Seasonally, the amount of carrageenan extracted from *E. denticulatum* varied little, from 53.06% dw to 66.03% dw [46]. During the alga’s juvenile phase in January and February, polysaccharide production is slightly lower, as the alga retains many growth nutrients. Yields are fairly constant from this month onwards, but increase slightly in April and May as the algae reach maturity [33]. From May onwards, mature algae are ready to be harvested and the unharvested remains reproduce, multiply again and become increasingly thalamus, stabilizing yields. Polysaccharide levels increase as *E. denticulatum* develops [46].

Galactose (over 40% TS), followed by glucose, glucuronic acid and xylose were identified in high monosaccharide contents. Some unidentified charged carbohydrates were detected with retention times close to those of glucuronic acid. Galacturonic acid could be present in *E. denticulatum* as it has been found in *S. chordalis*, *Gigartinale* and *K. alvarezii* [94,96]. It was found that the total monosaccharide content in the 2021 summer season ranged from 79.14 ± 1.65 to 81.74 ± 1.64% TS (February and October months, respectively) and in winter, these contents ranged from 81.20 ± 5.12 to 84.83 ± 3.85% TS (August and September months, respectively). Thus, in 2022, the summer season ranged from 77.60 ± 4.53 to 86.30 ± 5.37% TS (November and October, respectively) and the winter season from 74.21 ± 4.43 to 81.33 ± 9.29% TS. 6.0 (August and September, respectively). These results confirm the seasonal variability of monosaccharide levels, with higher levels in 2021 than in 2022. Variability in abiotic factors (water temperature, salinity, precipitation) and environmental factors (nutrients, nitrogen, ammonium, mineral elements) in the species studied could explain this difference. Given that these elements are necessary for algae to strengthen their cell walls, in red algae carrageenan was found to be a sulfated galactan, resulting in significant amounts of sugars, 3,6-anhydrogalactose and the sulfated group in the species studied. Two monosaccharides account for over 80% of the sugars present in *E. denticulatum*: glucose and galactose. The predominance of galactose confirms the presence of carrageenans in *E. denticulatum* [52]. Glucose, which makes up floridean starch, cellulose and hemicellulose, represents the vast majority of sugars present in the alga, with quantities varying little over the season (summer and winter). The presence of carrageenan is confirmed by IR analysis (Figure 4). The study by Vandanjon et al. (2023) showed that *E. denticulatum* polysaccharides vary with the seasons [46].

#### 3.2.5. Amino Acids

The protein is one of the three macronutrients needed by humans. The other two are carbohydrates and fats. Total amino acid composition of *E. denticulatum* is over 2% AA. On the basis of growth or nitrogen balance, amino acids have traditionally been classified as nutritionally essential (indispensable) or non-essential (dispensable) for animals and humans [97]. The protein content of *E. denticulatum* ranged from 2.04 ± 0.64 to 7.31 ± 0.72% of algal dry weight, which is slightly lower than other studies by Naseri et al. 2019, compared with the red algae *Chondrus crispus* and *Sarcothalia crispata* (14.25% and 12.41%, respectively) [98]. The protein values of the algal samples studied are 5.95 ± 0.48 and 5.32 ± 3.05% dw, respectively, for August 2021 and 2022, which corresponds to the value found by Muraguri et al., 2016 at 5.06 ± 0.36% dw [84]. The highest levels of non-essential amino acids in *E. denticulatum* include aspartic acid, glutamic acid, alanine, serine, glycine and hydroxy-proline. Next come the essential amino acids, whose composition is as follows: leucine, isoleucine, threonine, valine, phenylalanine, lysine, methionine, histidine and tryptophan. Since humans and animals cannot synthesize essential amino acids [99], it may be worthwhile to contribute to this scourge with this species. The conditionally essential amino acids, including traces of glycine, arginine, proline, tyrosine and cystine, are found at lower levels. Even if the body is capable of producing CEAA, it may not produce enough amino acids [100,101]. In this case, amino acids must be supplied by the diet, and this species can supply them in the form of dietary supplements. Thus, according to Figure 3, the structure of free and protein amino acids in *E. denticulatum* is roughly similar to that of vegetables, rich in alanine, aspartic acid, glutamic acid and glycine, although the ratios between their contents are very different [102].

Thus, comparing the ratio in relation to the two collection seasons in August that the ratios between Σ EAA/Σ AA are 40.34% (2021) and 40.32% (2022), referring to the article published by Naseri et al. 2019 [98], the ratio between Σ EAA/Σ AA of *E. denticulatum* is 46.30%, for *Kappaphycus alvarezii* 43.3% and for *Chondrus crispus* 40.9%. Similarly, the Σ NEAA/Σ AA ratio of the alga studied (*E. denticulatum* from Madagascar) is 59.657% (2021) and 59.68% (2022), and that of Naseri et al. 2019 is 53.7%, 56.7% and 59.1% (*E. denticulatum*, *K. alvarezii* and *C. crispus* species, respectively). Therefore, the Σ EAA/Σ NEAA ratio of *E. denticulatum* from Madagascar is 67.63% (2021) and 67.55% (2022) while for Naseri et al. 2019 it is 86.2%, 76.4% and 69.2% (*E. denticulatum*, *K. alvarezii* and *C. crispus*, respectively). So, the observed values of the species studied are lower than Nasseri’s, and the closest species is *Chondrus crispus*. Meanwhile, the alga *E. denticulatum* falls within the range of the ratio (Σ EAA/Σ AA) of beef and soya proteins between 48% and 47% [103]. The quality of a protein is mainly related to its essential amino acid content and digestibility [104]. With such an interesting structure, it is obvious that this species of algae is recommended for nutritional intake.

### 3.3. Antioxidant Activity

Antioxidants are increasingly used to explain various pathological conditions and their therapeutic approach [105]. Oxidative stress is known to generate reactive oxygen species and reactive oxygen and nitrogen species [106]. IC_50_ values for carrageenan extract from *E. denticulatum* were between 2026,5 ± 1,2 and 3050.2 ± 2,2 μg.mL^−1^ in year 2021, and between 1904.0 ± 1.1 and 2875.6 ± 2.0 μg.mL^−1^ in 2022. The results obtained with carrageenan extract from *E. denticulatum* of Madagascar are therefore superior to those of studies carried out by other researchers [27,80], which are based on carotenoid extracts (*E. denticulatum*) and the other on polysaccharide extracts (*E. cottonii)* according to the article published by Balasubramaniam et al. 2020 and Teo et al. 2021. The antioxidant activity of crude extracts from the alga *E. denticulatum* is influenced by several factors, including pigments and other antioxidants [81]. Thus, the potential and possibility of antioxidant mechanisms of algae/polysaccharide extracts rest not only on their reduction capacity, but also on their ability to trap metal ions that catalyze oxidation phenomena [92,107,108,109,110]. Furthermore, the antioxidant activity of *E. denticulatum* varies seasonally, with peaks in summer, due to increased sunshine and temperatures. This season stimulates the production of polysaccharides and carotenoids, or the other dominant pigment in red algae, which help combat oxidative stress [79]. Conversely, winter, marked by a drop in light and temperatures, decreases this production, leading to lower antioxidant efficacy [81,111]. These results underline the potential of algae in the food and cosmetics industries, where natural antioxidants are sought after for their beneficial properties.

**Figure 5:**
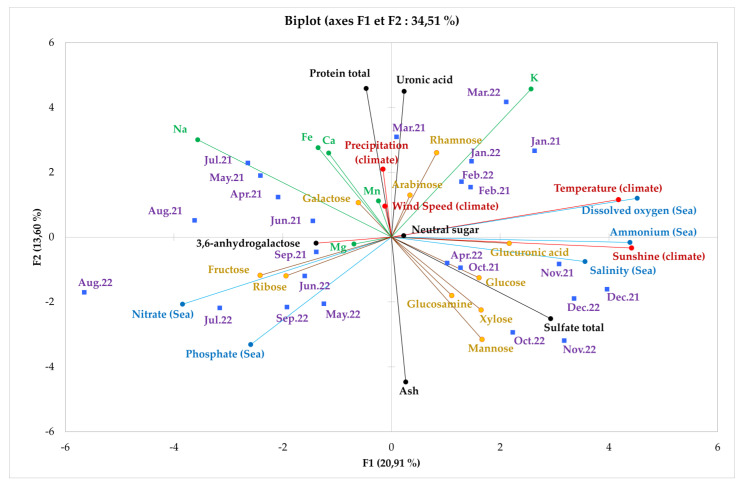
Principal component analysis (PCA) of the influence of seasonal variations of *E. denticulatum* on these abiotic and environmental factors and biochemical compositions during the year 2021–2022.

According to the PCA (Figure 5), nutrient compounds can show significant correlations with seasons. High concentrations of monosaccharides, as well as proteins, may interact with this activity. As far as abiotic factors are concerned, algae require oxygen and light for photosynthesis. They therefore need dissolved oxygen and participate in the nitrogen cycle to protect themselves from pathogens. There is therefore a strong correlation between protein, ammonium and dissolved oxygen. Han et al. 2015 showed that polysaccharides possessed potent antioxidant activities against DPPH, hydroxyl and superoxide anion radicals [79]. It has therefore been proposed that the possible antioxidant mechanism of polysaccharides involves the donation of hydrogen to break chain reactions and the free radical scavenging capacity resulting from the abstraction of anomeric hydrogen from the internal monosaccharide units of polysaccharides [96]. Consequently, the polysaccharide could be a good hydrogen donor capable of combining effectively with radicals and terminating the radical chain reaction [79].

### 3.4. Seasonality on Biochemical, Environmental and Abiotic Factors

To facilitate interpretation of this seasonal variability, a principal component analysis (PCA) was carried out. It was used to group together the information gathered during the analysis carried out. The PCA was applied on the basis of a table cross-referencing the individuals corresponding to the results obtained on *E. denticulatum* collected each month from 2021 to 2022 and the quantitative variables made up of the analytical results in biochemical composition, monosaccharide, mineral content and abiotic and environmental factors and antioxidant activity among 31 parameters to be analyzed (Figure 5). Axis correlation was 34.51%, with the F1 correlation axis being relatively low (20.91%) compared to the F2 axis (13.60%). According to Figure 5, the PCA was divided into four different regions.

Firstly, the F1 > 0 and F2 > 0 region, where mineral parameters such as potash (K) and the biochemical parameter uronic acid are the most correlated in this zone, and neutral sugar content is the most abundant in quantity which is rather close to the origin. Thus, the factors associated with this correlation are climate temperature and the dissolved oxygen content of seawater. The parameters of these correlations are focused on the observation variables from January to March of the years 2021 and 2022.

Next, in the F1 < 0 and F2 > 0 region, biochemical compositions such as total protein content, and mineral (sodium (Na), calcium (Ca) and iron (Fe)) and galactose contents of the monosaccharide composition are strongly correlated in this region. Similarly, the factors interacting with these correlations are precipitation and wind speed climate, and the variables observed are the months of April to August in the year 2021.

Then, for the region F1 < 0 and F2 < 0, the 3,6-anhydrogalactose content, the monosaccharide content (fructose and ribose) and the magnesium content (Mg) of the mineral composition are correlated in this region, where the nitrate and phosphate concentration of seawater is its interacting factor. The variables observed in this region are September 2021 and May to September 2022.

Finally, in the F1 > 0 and F2 < 0 region, biochemical compositions such as mineral matter (total ash) and total sulfate content are strongly correlated. So too are monosaccharide contents such as glucosamine, mannose, xylose, glucose and glucuronic acid, which are weakly correlated in this zone. Seawater salinity and sunshine are the interacting factors, as they are correlated during the months of April 2022 and October to December 2022.

The distribution of parameters as a function of observation variables reveals that these variables are linked to algal metabolism and are influenced by season. Abiotic and environmental factors have also been shown to have an impact on raw material growth metabolism. Furthermore, the seasonal distribution of these variables can be attributed to the protection system that the algae uses against the pathogen, as interactions between the plant and the pathogen are of two types: one is compatible susceptible and the other incompatible resistant [112].

*E. denticulatum* adapts its biochemical composition to seasonal variations, optimizing the production of bioactive compounds for growth, defense and survival. The four regions identified by the PCA show how environmental conditions influence key aspects of the alga’s metabolism, enabling it to thrive in constantly changing marine environments. These results confirm the importance of seasonality in regulating the physiological processes of this alga, and underline its potential role in biotechnological applications, notably in the production of high-value compounds for the food and pharmaceutical industries.

## 4. Materials and Methods

### 4.1. Materials

#### 4.1.1. Collection and Pretreatment of *Eucheuma Denticulatum* (N.L.Burman) Collins and Hervey 1917

The *E. denticulatum* algae were monthly harvested from the sea on the east coast of Madagascar, Sainte Marie Island, Analanjirofo region, Toamasina province (16°59′37.28″ S, 49°53′5.13″ E), during the years 2021 and 2022. Seaweed was washed in fresh water to remove salts and other debris, then traditionally dried on a bench in the shade for one to two weeks [93]. A total of 24 samples were recorded and stored in the dark for subsequent analysis [46].

#### 4.1.2. Seaweed Hydrolysis

To characterize the *E. denticulatum* raw material collected each month, the dried and crushed algae were subjected in triplicate to acid hydrolysis or to aqueous hydrolysis. Acid hydrolysis was carried out at 100 °C for 2 h with 10 mg of raw material in 5 mL of 1 M HCl. Then, 5 mL of 1M sodium hydroxide NaOH was added to neutralize the mixture. This solution is used for the determination of neutral sugars, proteins, uronic acids and 3,6-anhydrogalactose. For aqueous hydrolysis at 100 °C for 2 h, 10 mg was placed in the presence of 5 mL ultrapure water. Next, 5 mL ultrapure water was added to obtain a solution with a final concentration equal. This solution (1 mg.mL^−1^) was used for the determination of sulfate groups [83].

### 4.2. Analysis of Environmental and Abiotic Factors

Temperature, sunshine duration, wind speed and monthly precipitation were collected from the databases of Madagascar’s Directorate General of Meteorology (DGM). Salinity was measured in situ using oceanographic probes [113,114]. Dissolved oxygen was determined by the volumetric method [114,115]. For nitrate analysis, the method used is based on the determination of NO_2_^−^ ions obtained by quantitative reduction of NO_3_^−^ ions after passage of the sample through a copper-treated cadmium column [116]. The nitrate concentration is thus obtained by subtracting the nitrite concentration determined separately without reduction. Nitrate reduced to nitrite is determined spectrophotometrically (Beckman DU-64 UV/Vis Spectrophotometer, SpectraLab Scientific, Markham, ON, Canada) at 543 nm [117]. Analysis of ammoniacal or ammonium nitrogen has been referred to in ISO 7150/1 [118]. Thus, the seawater phosphate analysis method is based on the reaction of orthophosphate with molybdate, in the presence of antimony, to form the phosphomolybdic complex, which is then reduced by ascorbic acid to form a blue compound [61]. Colorimetric analysis is performed at a wavelength of 820 nm using a continuous flow autoanalyzer; the detailed analysis of this parameter was described in the written document Daniel in 2014 [119].

### 4.3. Biochemical Composition

The contents of proteins, uronic acids, neutral sugars, sulfates and 3,6-anhydrogalactose and mineral matter were analyzed to determine the biochemical composition of *E. denticulatum*, defined as percentages of each compound found in the total dry weight of the raw material and the results was expressed in percentages relative to dry weight (% dw). For the characterization of neutral sugars, proteins, uronic acids and 3,6-anhydrogalactose, the acid hydrolysis solution prepared previously in paragraph 4.1 was used, and for sulfate analysis, the aqueous extraction solution was used.

Neutral sugars were determined by the phenol-sulfuric acid colorimetric method described by Dubois [120], using anhydrous D-glucose (0–100 μg.mL^−1^) as a standard, the principle of which is based on the formation of a degradation product of sugars, under the action of a strong acid, into furfural derivatives. These chromogenic products are then condensed with phenol to obtain a chromophore [120].

The content of uronic acid was quantified by the meta-hydroxydiphenyl (MHDP) method [104] with glucuronic acid being the standard (0–100 μg.mL^−1^). The carboxylic groups of uronic acids are converted to methyl esters by acid hydrolysis to give 5-formylfuroic acids. These, complexed with MHDP and in the presence of sodium tetraborate, develop a pink coloration with a maximum absorbance at 525 nm [83,104].

The bicinchoninic acid (BCA) colorimetric method [121] with The Pierce BCA Protein Assay Kit (Fisher Scientific (Waltham, MA, USA), CAS: 7758-99-8) was used to measure protein content. For protein estimation, bovine serum albumin (0–100 μg.mL^−1^) was used as a standard. This assay is based on the Biuret reaction, which results in the reduction of Cu^2+^ ions to Cu^+^ by proteins in an alkaline medium. Cu^+^ then forms a complex with bicinchoninic acid, and this complex is formed with a maximum absorbance at 562 nm. 

The sulfate content was determined by the Azure A chloride method (Sigma-Aldrich, Darmstadt, Germany, Cas: 531-53-3) [48,83,88,122,123]. For this estimation, sulfated Dextran (17%) (0–100 μg.mL^−1^) was used as a standard. The principle of the reaction is that 3-amino-7-(dimethylamino)phenothizin-5-ium chloride (Azure A) complexes the sulfate groups. The chromophore complex emits a violet color measurable with a spectrophotometer at 535 nm.

Total ash analysis was determined gravimetrically following incineration of the samples during 2 h at 585 °C [83].

The colorimetric assay developed by Yaphe and Arsenault (1965) was used to assess the 3,6-anhydrogalactose composition of the various forms of *E. denticulatum*. The determination of 3,6-anhydrogalactose is based on the Seliwanoff reaction. In an acidic environment at 80 °C, cetoses (fructose or 3,6-anhydrogalactose) dehydrate faster than aldoses (most sugars). Acid hydrolysis of polysaccharides and oligosaccharides produces simple sugars, then furfural derivatives such as furfural. Furfural then interacts with resorcinol to create a condensed chromophore that reflects red and absorbs at a maximum wavelength of 480 nm. The analysis procedures are described in the article published by Yaphe and Arsenault (1965) and with a brief modification in Anne-Sophie Burlot’s 2016 thesis [83,124].

### 4.4. Analysis of Monosaccharide Composition

The monosaccharide composition of the samples was determined by high-performance anion exchange chromatography (HPAEC) with pulsed amperometric detection (PAD) (Thermo Dionex, Sunnyvale, CA, USA); the method was mirrored on the articles of Magdugo et al., 2020 and Pliego-Cortés et al., 2019 [16,125] but with a brief modification. The carrageenan extract powder (4 mg dry matter) was subjected to acid hydrolysis for 48 h at 100 °C using 110 μL of 1 M HCl and 1 mL ultrapure water in a flame-sealed glass ampoule. The mixture was then neutralized with 110 μL of 1 M NaOH and 780 μL of ultrapure water containing deoxyribose (internal standard) to a final concentration of 50 ppm. All samples were centrifuged for 10 min at 10,000 rpm, then the supernatant was collected and introduced into the vial. Details of the elution program can be found in Pliego-Cortés et al., 2019 [125]. Monosaccharides were identified and quantified on the basis of their standard curves at different concentrations (1.95–125 ppm), including deoxyribose, fructose, rhamnose, arabinose, glucosamine, galactose, glucose, mannose, xylose, fructose, ribose and glucuronic acid. Results were expressed in percentage of total sugar content (% TS).

### 4.5. Analysis of Mineral Compositions by Flame Atomic Absorption Spectrophotometry

The main mineral elements such as Ca, Mg, Na and K as macroelements as well as the trace elements Fe, Zn, Cu and Mn were determined using a PerkinElmer AAnalyst 200 atomic absorption spectrophotometer (AAS) equipped with a single hollow cathode lamp for each element and an air-acetylene burner. Crude samples (200 mg) of *E. denticulatum* dried algal powder were placed in digestion vessels with 1.3 mL 1 M HCl, and 18.7 mL ultrapure water was added (final volume is 20 mL after dilution) to give a final sample concentration of 10 mg.mL^−1^. The sample was then incubated for 48 h at 118 °C. Samples were filtered (0.22 µm, Minisart High Flow; Sartorius Stedim Biotech, Göttingen, Germany) prior to analysis. Filtered samples were diluted with distilled water to obtain a concentration of 1 mg.mL^−1^. Quantification was performed using element-dependent standard ranges [16].

### 4.6. Amino Acid Composition

For the determination of the amino acid profile of *E. denticulatum*, the in-house method ACIDAM 96/09 was chosen for the determination of total amino acids, approved by EC regulation 152/2009-SN of 27 January 2009 [126]. Free amino acids were determined and extracted with dilute hydrochloric acid; co-extracted macromolecules were precipitated with sulfosalicylic acid and filtered. The filtered solution was adjusted to pH 2.20. Amino acids were separated by ion exchange chromatography and analyzed by reaction with ninhydrin and photometric detection at 570 nm. Cysteine must be oxidized to cysteic acid and methionine to methionine sulfone before hydrolysis. Tyrosine must be analyzed from the hydrolysate of a non-oxidized sample. All other amino acids can be analyzed from oxidized or non-oxidized samples. The TRYPTO 95 in-house method has been adapted for the determination of total tryptophan [126]. The sample is hydrolyzed in alkaline medium with a saturated barium hydroxide solution and heated to 110 °C for twenty hours. After hydrolysis, the internal standard is added. For the determination of free tryptophan, the sample is extracted under conditions of moderate acidity in the presence of the internal standard. Tryptophan and the internal standard in the hydrolysate or extract are determined by liquid chromatography (LC) using a fluorometric detector. Results are expressed in relative percentage of algae dry weight in amino acids (% AA).

### 4.7. Carrageenan Extraction

Carrageenan from *E. denticulatum* was extracted using the classic hot extraction method [46]. Briefly, dry algae (around 80–100 g of dry algal weight) were depigmented with a 300 mL solution of acetone ≤ 99.8% (Fisher Scientific, Loughborough, UK, CAS: 67-64-1) for 4 h in a Soxhlet mount, followed by the same method in absolute ethanol ≤ 99 (Fisher Scientific, Loughborough, UK, CAS: 64-17-5). Depigmented algae were then extracted in 1.5 L of distilled water at 80 °C in a Thermomix for 4 h. The resulting solutions were then filtered through a cloth and the algae residues were re-extracted with distilled water in a Thermomix for 4 h at 80 °C. The filtrates obtained were precipitated separately overnight (in a cold room at 4 °C overnight) with 99% absolute ethanol and stirred gently with a magnetic stirrer (the volume of ethanol was double the volume of filtrate) and 4 teaspoons of sodium chloride, (Carlo Erba, Milan, Italy, CAS: 7647-14-5) were added. We then filtered the solution and the resulting solids were frozen at −80 °C for 1 h, freeze-dried, ground and stored in a dry place.

### 4.8. Fourier-Transform Infrared Spectroscopy of Carrageenan Extracts

Fourier-transform infrared (FTIR) spectra were recorded using a Nicolet iS5 FTIR spectrometer (Thermo Scientific, Waltham, MA, USA) equipped with an iD7 ATR module with a diamond crystal. Measurement and comparison of dried powders with an ι-carrageenan standard (Sigma commercial grade) were carried out to study the polysaccharides of *E. denticulatum*. All spectra resulted from 16 scans and a resolution of 4 (0.482 cm^−1^). OMNIC 9.2.86 software (Thermo Scientific, Waltham, MA, USA) was used for data acquisition (absorbance mode). A background reference is obtained before each sample measurement. All the samples were recorded in triplicates [46,127].

### 4.9. Antioxidant Activity of Carrageenan Extracts

For the radical scavenging activity of the antioxidant 2,2-diphenyl-1-picrylhydrazyl (DPPH), the method chosen was referenced by the articles cited below but with a brief modification [16,29,30,50,128,129]. A 0.1 mg.mL^−1^ solution of DPPH (0.25 mM) (Sigma-Aldrich, Darmstadt, Germany, Cas: 1898-66-4) was prepared with methanol, stored at 4 °C and protected from light and UV light. Standard solutions of butylated hydroxyanisole (BHA) (Sigma-Aldrich, Darmstadt, Germany, CAS: 128-37-0) and butylated hydroxytoluene (BHT) (Sigma-Aldrich, Darmstadt, Germany, CAS: 25013-16-5) at different concentrations ranging from 0 to 25 μg.mL^−1^ by methanol dilution (Sigma-Aldrich, Darmstadt, Germany, CAS: 67-56-1) were tested as positive controls. To obtain a range of BHA/BHT samples, dilute in methanol or MilliQ water up to 5 mL in a red-capped hemolysis tube. An initial wide range from 0 to 2000 μg.mL^−1^ sample is taken to determine the IC_50_. This range is cascaded on 14 from the stock solution. A second, tighter range is then run around the IC_50_ value to obtain a straight line with equation. The extracts of *E. denticulatum* algal carrageenan for analysis were prepared at a concentration of 1 mg.mL^−1^ in water. The sample solutions were diluted at the time of testing to a concentration of between 0 and 2500 µg.mL^−1^. Test solutions were added to the wells in triplicate with 100 µL of the total sample diluted in water, topped up with 100 µL of DPPH reagent. After shaking, the microplate was incubated at 40° C for 30 min before being read at 517 nm using a Thermo Scientific Multiscan Go UV-vis instrument (Thermo Scientific, Waltham, MA, USA). The scavenging capacity of the DPPH radical was calculated using the equation that has been described by Boulho et al. 2017 [34]. The scavenging capacity of the DPPH radical was calculated using the equation below. The IC50%, corresponding to the concentration sufficient to obtain 50% of the maximum trapping capacity of the samples, was determined on the basis of the regression obtained from the dose curve of the antiradical activity.

**Trapping % = [(A_Control_ − A_sample_)/A_Control_] × 100** (1)

where **A_Control_**: absorbance of the positive control of the reaction, and **A_sample_**: absorbance obtained by mixing with the test sample.

### 4.10. Statistical Analysis

For assays and other analyses, results are expressed as mean ± standard deviation (n = 3). Statistical analyses were performed using XLSTAT version 2024 software. One-way ANOVAs were performed, with the smallest difference at a significance level of 5% (*p* < 0.05) [130] and ANOVA analysis of variance. Principal component analysis (PCA) was carried out using the same software (XLSTAT), whose analysis method is an extremely powerful tool for compressing and synthesizing the information obtained and correlating the variables in relation to the selected observations [131].

## 5. Conclusions

To conclude, the species studied exhibit varying nutritional values and distinct biological and biochemical activities influenced by seasonal variability. The high content of neutral sugars and minerals in *E. denticulatum* during the summer months highlights its significant nutritional value, offering a rich source of amino acids, minerals and glucose. This alga’s leguminous structure contributes to its nutritional profile. During winter and in transitional seasons, it is notably rich in minerals, sulfate and galactose compounds. This study underscores the impact of both biotic and abiotic factors on the alga’s growth, significantly influencing the seasonal fluctuations in its biochemical composition. These factors determine the concentrations of key components that make *E. denticulatum* valuable for nutraceutical applications. Overall, this study highlights the complex interplay between environmental conditions and the biochemical properties of *E. denticulatum*, reinforcing its importance as a nutritionally rich and biologically active species.

## Figures and Tables

**Table 1:** Biochemical composition (% dw) of *Eucheuma denticulatum* crude fractions.

	Ash	Neutral Sugar	Uronic Acid	Protein Total	Sulfate Total	3,6-Anhydrogalactose	Total (% dw)
**January 2021**	22.8 ± 0.8	41.0 ± 0.4	3.084 ± 0.005	5.8 ± 0.4	6.4 ± 0.5	9.392 ± 0.009	88.38 ± 2.08
**January 2022**	24.7 ± 3.2	36.0 ± 0.1	2.063 ± 0.001	6.3 ± 0.4	9.1 ± 0.2	7.917 ± 0.002	86.05 ± 3.90
**February 2021**	23.0 ± 1.9	31.8 ± 0.1	1.963 ± 0.001	7.0 ± 0.6	5.1 ± 0.2	10.442 ± 0.001	79.13 ± 2.75
**February 2022**	24.0 ± 2.0	28.6 ± 0.1	2.102 ± 0.001	5.8 ± 0.4	3.7 ± 0.1	8.442 ± 0.003	72.43 ± 2.47
**March 2021**	23.3 ± 1.1	32.8 ± 0.1	3.004 ± 0.006	6.0 ± 1.0	4.30 ± 0.07	12.654 ± 0.003	82.01 ± 2.18
**March 2022**	25.2 ± 1.4	34.52 ± 0.03	2.436 ± 0.004	5.4 ± 0.6	4.23 ± 0.07	8.638 ± 0.002	80.43 ± 2.11
**April 2021**	26.2 ± 4.0	34.6 ± 0.1	1.516 ± 0.001	5.1 ± 0.2	4.5 ± 0.2	10.605 ± 0.001	82.52 ± 3.97
**April 2022**	38.5 ± 0.6	28.1 ± 0.1	2.106 ± 0.001	4.4 ± 1.3	6.2 ± 0.1	11.130 ± 0.002	90.42 ± 2.06
**May 2021**	21.4 ± 0.3	35.3 ± 0.1	1.738 ± 0.002	7.3 ± 0.7	5.4 ± 0.1	8.786 ± 0.001	79.92 ± 1.19
**May 2022**	23.7 ± 0.4	37.87 ± 0.05	1.987 ± 0.001	4.1 ± 3.1	8.8 ± 0.1	10.261 ± 0.001	86.76 ± 3.65
**June 2021**	24.3 ± 0.7	29.1 ± 0.1	1.978 ± 0.003	5.3 ± 0.4	3.93 ± 0.08	11.064 ± 0.021	75.60 ± 1.31
**June 2022**	28.3 ± 1.2	37.70 ± 0.01	1.592 ± 0.001	3.4 ± 2.4	5.9 ± 0.1	11.212 ± 0.001	88.10 ± 3.76
**July2021**	23.2 ± 2.2	28.93 ± 0.04	2.428 ± 0.001	6.0 ± 0.2	3.93 ± 0.04	7.344 ± 0.001	71.73 ± 2.49
**July 2022**	37.2 ± 3.6	33.9 ± 0.1	1.157 ± 0.002	3.2 ± 2.2	4.37 ± 0.02	8.098 ± 0.006	87.96 ± 5.84
**August 2021**	21.8 ± 0.9	26.3 ± 0.1	1.618 ± 0.001	6.0 ± 0.5	3.7 ± 0.1	8.048 ± 0.001	67.42 ± 1.53
**August 2022**	35.4 ± 4.1	31.5 ± 0.1	1.685 ± 0.002	5.3 ± 3.1	4.82 ± 0.12	11.655 ± 0.001	90.33 ± 7.31
**September 2021**	28.4 ± 5.0	37.4 ± 0.1	2.471 ± 0.003	4.8 ± 0.9	5.12 ± 0.07	11.655 ± 0.008	89.87 ± 5.93
**September 2022**	38.8 ± 2.9	36.10 ± 0.02	1.614 ± 0.001	3.1 ± 2.6	4.2 ± 0.1	9.589 ± 0.001	93.36 ± 5.68
**October 2021**	24.1 ± 0.3	37.6 ± 0.1	1.592 ± 0.001	5.4 ± 0.5	4.30 ± 0.05	7.622 ± 0.001	80.62 ± 0.86
**October 2022**	39.0 ± 1.8	37.05 ± 0.01	1.142 ± 0.001	4.4 ± 2.8	8.7 ± 0.5	8.475 ± 0.001	98.65 ± 5.05
**November 2021**	20.2 ± 6.8	31.78 ± 0.04	1.368 ± 0.001	5.8 ± 0.7	8.7 ± 0.2	10.638 ± 0.002	78.47 ± 7.83
**November 2022**	39.5 ± 0.3	29.4 ± 0.1	1.642 ± 0.002	2.6 ± 1.6	1.0 ± 0.3	7.753 ± 0.006	90.73 ± 2.19
**December 2021**	29.2 ± 1.6	31.1 ± 0.1	1.262 ± 0.001	5.3 ± 0.4	14.5 ± 0.3	8.966 ± 0.003	90.27 ± 2.44
**December 2022**	39.5 ± 0.1	29.96 ± 0.01	1.579 ± 0.002	2.1 ± 0.6	6.01 ± 0.21	11.081 ± 0.007	90.12 ± 0.91

Data are means ± SD (n = 3). Significant GLM difference per line with a threshold of (*p* ≤ 0.05).

**Table 2:** *Eucheuma denticulatium* carrageenan yields collected from 2021 to 2022 (expressed in % dw).

	*Eucheuma denticulatium* (Hot Extraction) *	
Months	Carrageenan Yield (% dw) 2021	Carrageenan Yield (% dw) 2022
**January**	53.7 ± 3.8 ^ab^	53.1 ± 6.5 ^a^
**February**	55.1 ± 4.3 ^a^	54.6 ± 2.0 ^ab^
**March**	57.2 ± 2.8 ^a^	61.2 ± 2.0 ^ab^
**April**	57.7 ± 1.6 ^a^	66.0 ± 1.6 ^ab^
**May**	60.4 ± 2.5 ^ab^	63.0 ± 1.0 ^ab^
**June**	59.1 ± 2.3 ^b^	61.2 ± 2.2 ^a^
**July**	61.7 ± 1.0 ^a^	60.8 ± 2.1 ^b^
**August**	59.7 ± 0.7 ^a^	58.7 ± 1.6 ^ab^
**September**	60.1 ± 1.6 ^ab^	58.2 ± 3.0 ^b^
**October**	60.5 ± 1.8 ^a^	60.9 ± 2.1 ^a^
**November**	61.2 ± 0.4 ^a^	60.1 ± 1.9 ^ab^
**December**	61.7 ± 0.5 ^a^	61.0 ± 0.5 ^a^

* Hot extraction in a Thermomix at 80 °C for 4 h (LBCM, France). Evolution of extracted carrageenan yield for the years 2021 and 2022. Significant changes are indicated by letters other than a and b, with a *p*-value < 0.05 for sample size n = 3.

**Table 3:** Monosaccharide composition of carrageenan extracts of *Eucheuma denticulatum* algae from Madagascar in 2021–2022, expressed in % TS.

	Ara	Rha	Glc	Gal	Glu	Man	Xyl	Fru	Rib	Glu.Aca	Total (% TS)
**January 2021**	0.129 ± 0.003	0.353 ± 0.001	0.022 ± 0.002	49.5 ± 1.7	2.12 ± 0.02	0.002 ± 0.001	1.1 ± 0.1	0.002 ± 0.002	0.040 ± 0.003	1.17 ± 0.01	79.4 ± 1.8
**January 2022**	0.131 ± 0.003	0.353 ± 0.001	0.022 ± 0.001	49.3 ± 1.6	2.13 ± 0.02	0.001 ± 0.001	1.0 ± 0.1	0.003 ± 0.002	0.042 ± 0.003	1.17 ± 0.01	79.1 ± 1.6
**February 2021**	0.135 ± 0.002	0.63 ± 0.02	0.02 ± 0.01	50.1 ± 1.5	2.01 ± 0.03	0.002 ± 0.001	0.99 ± 0.02	0.003 ± 0.001	0.036 ± 0.006	1.24 ± 0.04	79.9 ± 1.5
**February 2022**	0.10 ± 0.04	0.363 ± 0.002	0.002 ± 0.003	50.1 ± 0.4	2.495 ± 0.000	0.002 ± 0.001	0.8 ± 0.4	0.013 ± 0.002	0.021 ± 0.004	0.6 ± 0.6	79.5 ± 0.8
**March 2021**	0.14 ± 0.01	0.371 ± 0.003	0.012 ± 0.003	52.4 ± 1.5	1.6 ± 0.5	0.002 ± 0.000	0.8 ± 0.1	0.003 ± 0.000	0.09 ± 0.04	1.30 ± 0.08	81.7 ± 1.9
**March 2022**	0.158 ± 0.004	0.39 ± 0.01	0.026 ± 0.003	51.7 ± 0.5	1.9 ± 0.6	0.011 ± 0.004	0.7 ± 0.1	0.003 ± 0.001	0.16 ± 0.01	1.39 ± 0.02	81.3 ± 0.6
**April 2021**	0.157 ± 0.003	0.391 ± 0.003	0.02 ± 0.01	51.7 ± 0.5	2.0 ± 0.5	0.010 ± 0.004	0.7 ± 0.1	0.003 ± 0.001	0.16 ± 0.01	1.40 ± 0.01	81.4 ± 0.5
**April 2022**	0.153 ± 0.004	0.385 ± 0.005	0.002 ± 0.001	54.4 ± 3.3	2.4 ± 0.4	0.07 ± 0.02	1.0 ± 0.1	0.004 ± 0.003	0.05 ± 0.02	1.34 ± 0.07	84.8 ± 3.9
**May 2021**	0.160 ± 0.004	0.368 ± 0.001	0.016 ± 0.004	50.6 ± 4.6	2.5 ± 0.3	0.13 ± 0.09	1.1 ± 0.1	0.002 ± 0.001	0.06 ± 0.07	1.2 ± 0.2	81.2 ± 5.1
**May 2022**	0.145 ± 0.005	0.37 ± 0.03	0.011 ± 0.003	50.6 ± 1.4	2.4 ± 0.2	0.13 ± 0.02	1.08 ± 0.03	0.007 ± 0.006	0.23 ± 0.06	1.28 ± 0.06	81.3 ± 1.5
**June 2021**	0.15 ± 0.01	0.35 ± 0.01	0.010 ± 0.001	51.0 ± 1.5	2.5 ± 0.2	0.119 ± 0.004	1.08 ± 0.01	0.02 ± 0.02	0.19 ± 0.06	1.31 ± 0.06	81.7 ± 1.6
**June 2022**	0.15 ± 0.01	0.38 ± 0.01	0.020 ± 0.002	47.1 ± 7.9	2.2 ± 0.2	0.13 ± 0.022	1.0 ± 0.1	0.001 ± 0.001	0.32 ± 0.01	1.2 ± 0.2	77.5 ± 8.4
**July2021**	0.15 ± 0.02	0.374 ± 0.001	0.011 ± 0.002	52.2 ± 4.1	2.3 ± 0.8	0.002 ± 0.001	0.90 ± 0.04	0.011 ± 0.004	0.14 ± 0.09	1.42 ± 0.03	82.5 ± 3.4
**July 2022**	0.13 ± 0.02	0.34 ± 0.03	0.03 ± 0.01	47.8 ± 4.4	2.1 ± 0.3	0.09 ± 0.03	1.04 ± 0.08	0.001 ± 0.001	0.11 ± 0.04	1.45 ± 0.36	78.1 ± 4.7
**August 2021**	0.25 ± 0.16	2.7 ± 0.1	0.02 ± 0.01	49.5 ± 2.4	2.6 ± 0.3	0.06 ± 0.06	1.0 ± 0.1	0.002 ± 0.001	0.12 ± 0.05	1.3 ± 0.1	82.7 ± 2.7
**August 2022**	0.17 ± 0.03	0.32 ± 0.05	0.024 ± 0.003	49.1 ± 6.0	2.5 ± 0.3	0.03 ± 0.01	1.2 ± 0.1	0.003 ± 0.001	0.18 ± 0.01	1.4 ± 0.2	79.9 ± 6.6
**September 2021**	0.155 ± 0.003	0.38 ± 0.06	0.019 ± 0.001	48.3 ± 0.1	2.5 ± 0.1	0.06 ± 0.03	1.06 ± 0.04	0.004 ± 0.002	0.20 ± 0.07	1.30 ± 0.03	79.0 ± 0.1
**September 2022**	0.14 ± 0.01	0.40 ± 0.04	0.020 ± 0.010	50.9 ± 9.2	2.1 ± 0.2	0.12 ± 0.02	1.0 ± 0.2	0.003 ± 0.002	0.09 ± 0.04	1.3 ± 0.2	81.0 ± 9.7
**October 2021**	0.15 ± 0.01	0.36 ± 0.02	0.024 ± 0.004	49.0 ± 7.5	2.32 ± 0.05	0.100 ± 0.002	1.10 ± 0.05	0.009 ± 0.001	0.09 ± 0.03	1.28 ± 0.06	79.4 ± 7.6
**October 2022**	0.16 ± 0.02	0.36 ± 0.02	0.017 ± 0.003	43.2 ± 3.6	2.4 ± 0.5	0.001 ± 0.000	1.0 ± 0.2	0.11 ± 0.10	1.4 ± 1.2	0.6 ± 0.2	74.2 ± 4.4
**November 2021**	0.14 ± 0.02	0.43 ± 0.01	0.03 ± 0.01	52.3 ± 9.2	1.2 ± 0.2	0.002 ± 0.002	0.88 ± 0.06	0.009 ± 0.002	0.009 ± 0.001	1.3 ± 0.2	81.3 ± 9.3
**November 2022**	0.14 ± 0.01	0.41 ± 0.02	0.02 ± 0.01	55.2 ± 4.7	2.7 ± 0.3	0.09 ± 0.01	1.2 ± 0.2	0.003 ± 0.002	0.020 ± 0.009	1.5 ± 0.2	86.3 ± 5.4
**December 2021**	0.15 ± 0.01	0.36 ± 0.01	0.02 ± 0.01	47.1 ± 4.3	2.5 ± 0.1	0.15 ± 0.03	1.01 ± 0.03	0.002 ± 0.001	0.03 ± 0.02	1.3 ± 0.1	77.6 ± 4.6
**December 2022**	0.13 ± 0.06	0.39 ± 0.01	0.021 ± 0.004	48.0 ± 5.3	2.5 ± 0.6	0.031 ± 0.005	1.07 ± 0.15	0.002 ± 0.001	0.09 ± 0.07	1.6 ± 0.4	78.8 ± 6.2

Data are means ± SD (n = 3). Significant GLM difference per line with a threshold of (*p* ≤ 0.05), meaning Ara = Arabinose; Rha = Rhamnose; Glc = Glucosamine; Gal = Galactose; Glu = Glucose; Man = Mannose; Xyl = Xylose; Fru = Fructose; Rib = Ribose; Glu.Ac = Glucuronic acid.

**Table 4:** Summary of antioxidant activity, scavenging test (DPPH) of *Eucheuma denticulatum* carrageenan extracts from Madagascar in 2021–2022.

Antioxidant Inhibition Activity of *Eucheuma denticulatum*		
	IC_50_ (µg.mL^−1^) 2021	IC_50_ (µg.mL^−1^) 2022
**Standard BHA**	8.2 ± 0.7	
**Standard BHT**	13.5 ± 0.6	
**January**	2770.3 ± 1.2 ^a^	2656.4 ± 1.7 ^ab^
**February**	3050.2 ± 2.2 ^abc^	2875.6 ± 2.0 ^ab^
**March**	3049.2 ± 3.5 ^abc^	2744.1 ± 1.8 ^ab^
**April**	2709.0 ± 1.8 ^ab^	2596.8 ± 1.7 ^ab^
**May**	2342.4 ± 1.4 ^ab^	2530.5 ± 1.6 ^ab^
**June**	2263.4 ± 1.3 ^a^	2151.2 ± 1.2 ^a^
**July**	2116.1 ± 1.2 ^a^	2003.8 ± 1.4
**August**	2026.5 ± 1.2 ^a^	1904.0 ± 1.1
**September**	2659.9 ± 1.7 ^ab^	2234.7 ± 1.4 ^ab^
**October**	2356.7 ± 1.5 ^ab^	2233.7 ± 1.7 ^ab^
**November**	2715.0 ± 1.8 ^abc^	2592.6 ± 1.7 ^ab^
**December**	2596.8 ± 1.7 ^ab^	2484.5 ± 1.5 ^ab^

IC_50_ corresponds to the concentration sufficient to obtain 50% of maximum trapping capacity; BHA: Butylated hydroxyanisole; BHT: Butylhydroxytoluene. Data are means ± s.e.m. (n = 3). GLM difference (one-sample test of variance/two-tailed test) significant per column with a threshold of *p* ≤ 0.05). Lower-case letter denotes significance threshold at *p* < 0.05.

## Data Availability

The data illustrating this article are available in this link https://drive.google.com/file/d/1HH87Ny5x1J8yUK4c-SMh32mB4ogtJyL7/view?usp=sharing.
